# The Childhood Liver Disease Research Network's prospective characterization of pediatric primary sclerosing cholangitis

**DOI:** 10.1097/HC9.0000000000001049

**Published:** 2026-09-18

**Authors:** Cara L. Mack, Alexander G. Miethke, Kieran Hawthorne, Lisa Henn, Simon Horslen, Estella M. Alonso, Nitika A. Gupta, Shikha S. Sundaram, Rohit Kohli, Molly Bozic, Mark Deneau, Stephen Guthery, Kathleen M. Loomes, Pamela L. Valentino, Mary Elizabeth M. Tessier, Benjamin Shneider, John C. Magee, Saul Karpen, Phillip Rosenthal, Kasper Wang, Amanda Ricciuto, Ronald J. Sokol, Binita M. Kamath

**Affiliations:** 1 Division of Pediatric Gastroenterology, Hepatology & Nutrition, Department of Pediatrics, Medical College of Wisconsin, Children’s Wisconsin, Milwaukee, Wisconsin, USA; 2 Division of Gastroenterology, Hepatology and Nutrition, Department of Pediatrics, Cincinnati Children’s Hospital Medical Center, University of Cincinnati College of Medicine, Cincinnati, Ohio, USA; 3 Arbor Research Collaborative for Health, Ann Arbor, Michigan, USA; 4 Division of Pediatric Gastroenterology, Hepatology and Nutrition, Department of Pediatrics, UPMC Children’s Hospital of Pittsburgh, University of Pittsburgh School of Medicine, Pittsburgh, Pennsylvania, USA; 5 Division of Gastroenterology, Hepatology and Nutrition, Department of Pediatrics, Ann & Robert H. Lurie Children’s Hospital of Chicago, Northwestern University Feinberg School of Medicine, Chicago, Illinois, USA; 6 Division of Gastroenterology, Hepatology and Nutrition, Department of Pediatrics, Emory University School of Medicine, Atlanta, Georgia, USA; 7 Section of Gastroenterology, Hepatology and Nutrition, Department of Pediatrics, Children’s Hospital Colorado, University of Colorado School of Medicine, Aurora, Colorado, USA; 8 Division of Gastroenterology, Hepatology and Nutrition, Department of Pediatrics, Children’s Hospital Los Angeles, University of Southern California, Keck School of Medicine, Los Angeles, California, USA; 9 Division of Gastroenterology and Hepatology, Department of Pediatrics, Riley Hospital for Children, Indiana University School of Medicine, Indianapolis, Indiana, USA; 10 Division of Gastroenterology, Hepatology and Nutrition, Department of Pediatrics, Intermountain Primary Children’s Hospital, University of Utah, Salt Lake City, Utah, USA; 11 Division of Gastroenterology, Hepatology and Nutrition, Department of Pediatrics, Children’s Hospital of Philadelphia, Perelman School of Medicine at the University of Pennsylvania, Philadelphia, Pennsylvania, USA; 12 Division of Gastroenterology and Hepatology, Department of Pediatrics, Seattle Children’s Hospital, University of Washington School of Medicine, Seattle, Washington, USA; 13 Division of Gastroenterology, Hepatology and Nutrition, Department of Pediatrics, Texas Children’s Hospital, Baylor College of Medicine, Houston, Texas, USA; 14 Department of Surgery, Section of Transplant Surgery, University of Michigan Medical School, Ann Arbor, Michigan, USA; 15 Stravitz-Sanyal Institute for Metabolic Health and Liver Disease, Virginia Commonwealth University, Richmond, Virginia, USA; 16 Division of Gastroenterology, Hepatology and Nutrition, Department of Pediatrics, University of California San Francisco, San Francisco, California, USA; 17 Division of General and Thoracic Surgery, Hospital for Sick Children, University of Toronto, Toronto, Ontario, Canada; 18 Division of Gastroenterology, Hepatology and Nutrition, Department of Pediatrics, Hospital for Sick Children, University of Toronto, Toronto, Ontario, Canada

**Keywords:** autoantibody, autoimmune hepatitis, inflammatory bowel disease, SCOPE index, transient elastography

## Abstract

**Background:**

Primary sclerosing cholangitis (PSC) is a rare biliary fibrosing disease that leads to significant morbidity and the need for liver transplantation. The Childhood Liver Disease Research Network is conducting a prospective, observational longitudinal study to define the natural history of pediatric PSC. The aim of this report is to characterize the clinical phenotypes of the first 175 children enrolled in the PSC study and to determine correlations of specific phenotypes with clinical and laboratory characteristics.

**Methods:**

Data collected included a history of inflammatory bowel disease (IBD), autoimmune hepatitis (AIH), laboratories, autoantibodies, liver stiffness measurements (LSM), medications, and IBD activity. Comparison of phenotypes with/without IBD and/or AIH, and small-duct versus large-duct PSC was performed.

**Results:**

The median age at enrollment was 16.1 years, 60% were male, 78% had large-duct PSC, and the median duration of PSC was 2.2 years. The predominant phenotype was PSC/IBD, and 96% had quiescent/mild IBD. The entire cohort had evidence of mild liver disease based on biochemistries and LSMs; <10% had evidence of portal hypertension. Higher LSMs were associated with elevated liver biochemistries. There were no significant characteristics that differentiated the phenotypes; however, there was more frequent autoantibody positivity in PSC/IBD and higher LSMs in PSC/AIH overlap syndrome.

**Conclusions:**

This prospective cohort of pediatric PSC revealed mild liver disease in the majority. Laboratory biomarkers of liver injury and fibrosis were associated with LSM severity, suggesting LSM as a reliable tool to monitor PSC progression. This dataset encompasses a well-phenotyped cohort of children with PSC that will be followed prospectively for longitudinal assessment of progression of disease, as well as a model for stratification within future treatment trials.

Primary sclerosing cholangitis (PSC) is a rare disease with important public health implications, as it is a common indication for liver transplantation and is associated with a significantly increased risk of hepatobiliary and colorectal malignancies in adults. The prevalence of PSC in North America is estimated to be 10 per 100,000 in adults and 1.5 cases per 100,000 in children.^1^,^2^,^3^,^4^ Up to 80% of children diagnosed with PSC have concurrent inflammatory bowel disease (IBD), typically with colonic involvement. In participants with IBD, the prevalence rate of PSC is estimated to be ~5%–8%.^5^ Children with PSC have a variable course during the first few years following diagnosis, and the natural history of disease progression is poorly understood. A large retrospective study of children with PSC with a median age of presentation of 12 years demonstrated that event-free survival (free of portal hypertension, biliary complications, cholangiocarcinoma, transplant, or death) was 70% and 53% at 5 and 10 years, respectively.^6^ However, the contribution of comorbidities, including IBD and autoimmune hepatitis (AIH), to the progression of bile duct injury and associated fibrosis is poorly understood.

The Childhood Liver Disease Research Network (ChiLDReN), a multicenter North American consortium (13 sites) supported by the National Institute of Diabetes, Digestive and Kidney Diseases, is conducting a prospective observational and longitudinal study with the overarching goal of better defining the natural history and specific characteristics of PSC in childhood. ChiLDReN seeks to close knowledge gaps regarding phenotypes of PSC and to deepen the understanding of how IBD and AIH may impact the severity of PSC. The aim of this report is to characterize the clinical phenotypes of the first 175 children enrolled in the prospective PSC study and to determine correlations of specific phenotypes with clinical, laboratory, and histologic characteristics.

## METHODS

### Study population

Prospective enrollment baseline data and retrospective data at PSC diagnosis were analyzed from the ChiLDReN Prospective Observational Study of PSC in Children (clinicaltrials.gov ID# NCT04181138). Ethical approval for this study was obtained by the central IRB for the network, with ceding of authority by each site’s IRB. All research was conducted in accordance with both the Declarations of Helsinki and Istanbul. Written consent was given in writing by all participants/guardians, and assent of participants was obtained as per local institutional policies. Inclusion criteria were patients aged 2–25 years at the time of screening with either large-duct PSC based on MRCP criteria or small-duct PSC only, defined as liver histologic findings consistent with small-duct PSC and a normal MRCP at diagnosis. All research participants had the diagnostic MRCP or liver histology re-reviewed by a designated site radiologist or pathologist, respectively, to confirm the PSC diagnosis. Exclusion criteria included liver or bone marrow transplantation, causes of secondary sclerosing cholangitis (immunodeficiency, Langerhans cell histiocytosis, hemophagocytic lymphohistiocytosis, systemic lupus erythematosus, ischemic cholangitis, portal vein thrombosis with biliopathy, sinusoidal obstruction syndrome, and abdominal radiation vasculopathy), biliary atresia, history of biliary tract surgery or trauma, cardiac hepatopathy, sickle cell disease, genetic disorders involving the liver (cystic fibrosis, Caroli disease/congenital hepatic fibrosis, progressive familial intrahepatic cholestasis type 3, Wilson disease, glycogen storage disorders, alpha-1 antitrypsin deficiency), and concurrent pregnancy at the time of enrollment.

Select data that pertained to the original PSC, IBD, and/or AIH diagnoses before enrollment (MRCP findings, liver and intestinal histology findings, endoscopy findings, IBD activity scores, biochemical laboratories) were collected retrospectively. A clinical diagnosis of AIH was defined as the presence of all of the following criteria:^7^ (1) liver histology with lymphocytic interface hepatitis; (2) elevated IgG above the upper limit of normal (ULN); and (3) positive autoantibodies for anti-nuclear antibody (ANA), anti-actin/anti-smooth muscle antibody (ASMA), and/or liver kidney microsomal antibody (LKM). Small-duct PSC was defined histologically based on data collected at enrollment, which included demographics, laboratory parameters, physical exam, previous history of IBD, AIH, or other autoimmune diseases, medications, and IBD activity.

At enrollment, laboratory biochemistry results that were obtained for clinical purposes within 90 days of enrollment were collected. In addition, autoantibody testing (ANA, ASMA, LKM) and total IgG were considered research studies at enrollment, and participants were asked to have these additional laboratories obtained. Participants also underwent transient elastography (FibroScan, Echosens, Paris, France) to measure liver stiffness at enrollment.^8^

#### Study variables

(1) At enrollment, height and weight z-scores were calculated using SAS programs provided by the CDC based on growth charts (https://www.cdc.gov/growth-chart-training/hcp/computer-programs/sas.html); growth failure was defined as a z-score <−2 for either height or weight. (2) Alkaline phosphatase (ALP) scores were normalized using the age-specific and sex-specific z-score transformations.^9^ (3) The Sclerosing Cholangitis Outcomes in PEdiatrics (SCOPE) Index provides prognostic and risk-stratification information for pediatric PSC cases.^10^ The SCOPE indices at the time of PSC diagnosis and at study enrollment were calculated using total bilirubin, albumin, platelet count, GGT, and “large-duct involvement.” The SCOPE index predicts the risk of progression to liver transplant or death and the risk of development of portal hypertensive or hepatobiliary complications; risk is categorized as low (0–3 points), medium (4–5), or high (6–11).

#### Statistical analysis

Variables were summarized using frequency and percentages for categorical variables or median and range for continuous variables, in both tabular and graphical formats. Categorical variables were compared using chi-square tests unless one or more cells in the cross-tabulation had a count of 5 or less; in that case, we used the Fisher exact test. Continuous variables were compared between groups using the Wilcoxon rank sum test or the Kruskal–Wallis test in the case of more than 2 groups. *p*-Values were adjusted for multiple comparisons using the adaptive step-up Bonferroni method.^11^ All *p*-values within each table were corrected as 1 set, based on the number of comparisons within that table. All statistical analyses were performed using SAS Enterprise Guide 8.3 (Cary, NC).

## RESULTS

### Cross-sectional characterization of pediatric PSC cohort

**FIGURE 1:**
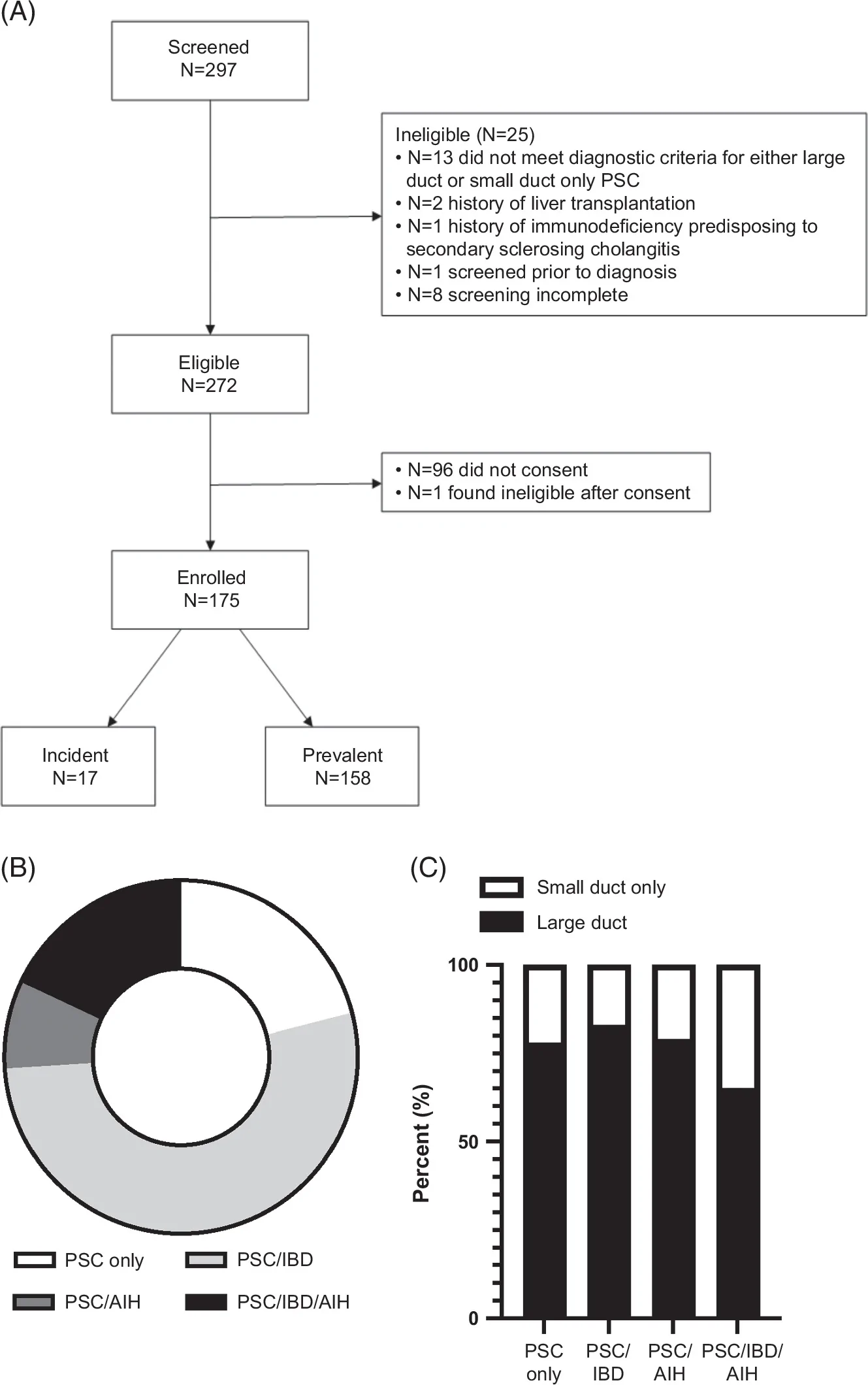
Cross-sectional characterization of pediatric PSC cohort. (A) Screening/enrollment flow diagram. Percentage frequency of (B) various PSC phenotypes and (C) large-duct and small-duct-only PSC based on a specific clinical phenotype. Abbreviations: AIH, autoimmune hepatitis; IBD, inflammatory bowel disease; PSC, primary sclerosing cholangitis.

Between December 1, 2021, and August 31, 2025, 297 research participants were screened, and 272 were found eligible. Ninety-six participants did not agree to consent, and therefore, the final number enrolled was 175 (Figure 1A). Ninety percent of participants had been previously diagnosed with PSC (“prevalent” cases), while 10% were newly diagnosed within 90 days of enrollment (“incident” cases). The median age at enrollment was 16.1 years (range 2.5–25.6), 60% were male, 87% White, and 92% non-Hispanic. The median length of time from original PSC diagnosis to enrollment was 2.2 years (range 0.1–14.5) (Table 1). Medication use at enrollment was noteworthy for frequent use of ursodeoxycholic acid (51%) and oral vancomycin (27%) (Supplemental Table S1, https://links.lww.com/HC9/C488). A previous diagnosis of concurrent IBD and/or AIH was reported in 74% and 34% at enrollment, respectively. The predominant PSC phenotype was PSC/IBD (53%), followed by PSC only (20%), PSC/IBD/AIH (18%), and PSC/AIH (8%) (Figure 1B). Other autoimmune diseases reported included celiac disease (5%), juvenile idiopathic arthritis (2%), psoriatic disease (2%), eosinophilic esophagitis (1%), and 1 case each of type 1 diabetes mellitus, thyroid disease, vitiligo, spondyloarthritis, autoimmune pancreatitis, and polyarteritis nodosa. With regard to family history of IBD or PSC in first-degree relatives, 2%–4% of participants had a parent with IBD, 4% had a sibling with IBD, and 1% had a sibling with PSC.

**TABLE 1:** Demographics at enrollment

	Total, N	n (%) or median (range)
Cohort		
Incident	175	17 (9.7%)
Prevalent		158 (90.3%)
Age at consent (years)	175	16.0 (2.5, 25.6)
Sex		
Male	172	103 (59.9%)
Female		69 (40.1%)
Race		
White	160	140 (87.5%)
Black or African American		11 (6.9%)
Asian		6 (3.8%)
Native American/Pacific Islander		1 (0.6%)
Multiracial		2 (1.3%)
Ethnicity		
Non-Hispanic	157	145 (92.4%)
Hispanic		12 (7.6%)
Duration of PSC (years)	168	2.2 (0.1, 14.5)
IBD at enrollment	168	124 (73.8%)
AIH at enrollment	131	45 (34.4%)
Diagnosis		
Large-duct PSC	175	136 (77.7%)
Small-duct-only PSC		39 (22.3%)

Abbreviations: AIH, autoimmune hepatitis; IBD, inflammatory bowel disease; PSC, primary sclerosing cholangitis.

The majority of participants had large-duct PSC (78%), and 22% had small-duct-only PSC. There was no significant difference in the frequency of small-duct-only PSC within the various PSC phenotypes; however, small-duct-only PSC was most prevalent in the PSC/IBD/AIH phenotype (36%) compared with other phenotypes (17%–21%) (Figure 1C). Review of *retrospective data related to the original PSC diagnosis* revealed the following MRCP findings of large-duct PSC: focal bile duct strictures (67%), beaded appearance of bile ducts (61%), pruning of distal biliary branches (36%), dominant stricture of the common bile duct (24%), and saccular dilation of bile duct(s) (11%). There were no significant differences in the frequency of specific diagnostic MRCP findings between PSC phenotypes; however, there was a higher prevalence of focal stricturing of bile ducts in the PSC/AIH phenotype (100% of PSC/AIH, 55%–69% in other phenotypes; *p*=0.07) (Supplemental Figure S1A, https://links.lww.com/HC9/C487; Supplemental Table S2, https://links.lww.com/HC9/C488). Diagnostic liver histology findings of small-duct-only PSC included periductal fibrosis/“onion skinning” (69%), bile duct epithelial injury (69%), ductular reaction (65%), neutrophils in bile ducts (49%), periductal edema (44%), and concentric periductal inflammation (44%). Among the 50 participants who had liver biopsies reviewed for the diagnosis of small-duct PSC, there were no significant differences in the frequency of specific liver histologic findings between PSC phenotypes, however concentric periductal inflammation, bile duct injury and neutrophils in bile ducts were most prevalent in the PSC/AIH phenotype (100% of PSC/AIH had these findings) (Supplemental Figure S1B, https://links.lww.com/HC9/C487; Supplemental Table S2, https://links.lww.com/HC9/C488).

### The severity of liver disease is mild in the majority of the pediatric PSC cohort

The severity of liver disease in PSC was measured based on retrospective data related to historical clinical manifestations, enrollment physical exam findings, laboratories, and liver stiffness measurements. Historical manifestations of liver disease severity included the presence of esophageal/gastric varices (7%), bacterial cholangitis requiring hospitalization (5%), and performance of endoscopic retrograde cholangiopancreaticogram (ERCP) (24%). Approximately 50% of those who had undergone ERCP had balloon dilatation with or without biliary stent placement. The SCOPE index at the *time of the PSC diagnosis* indicated 40% were in the low-risk category, 47% medium-risk, and 12% high-risk. Higher SCOPE risk scores *at diagnosis* were associated with significantly higher bilirubin, AST, ALT, GGT, AST/platelet ratio index (APRI), and FIB-4 at enrollment, based on linear regression modeling of a specific laboratory value and the SCOPE index, adjusted for the duration of PSC (time from diagnosis to enrollment) (Supplemental Table S3, https://links.lww.com/HC9/C488). In contrast, the SCOPE risk index at enrollment reflected milder disease severity, with 72% in the low-risk category, 23% medium-risk category, and 5% high-risk category. There were no cases of cholangiocarcinoma in this cohort.

**FIGURE 2:**
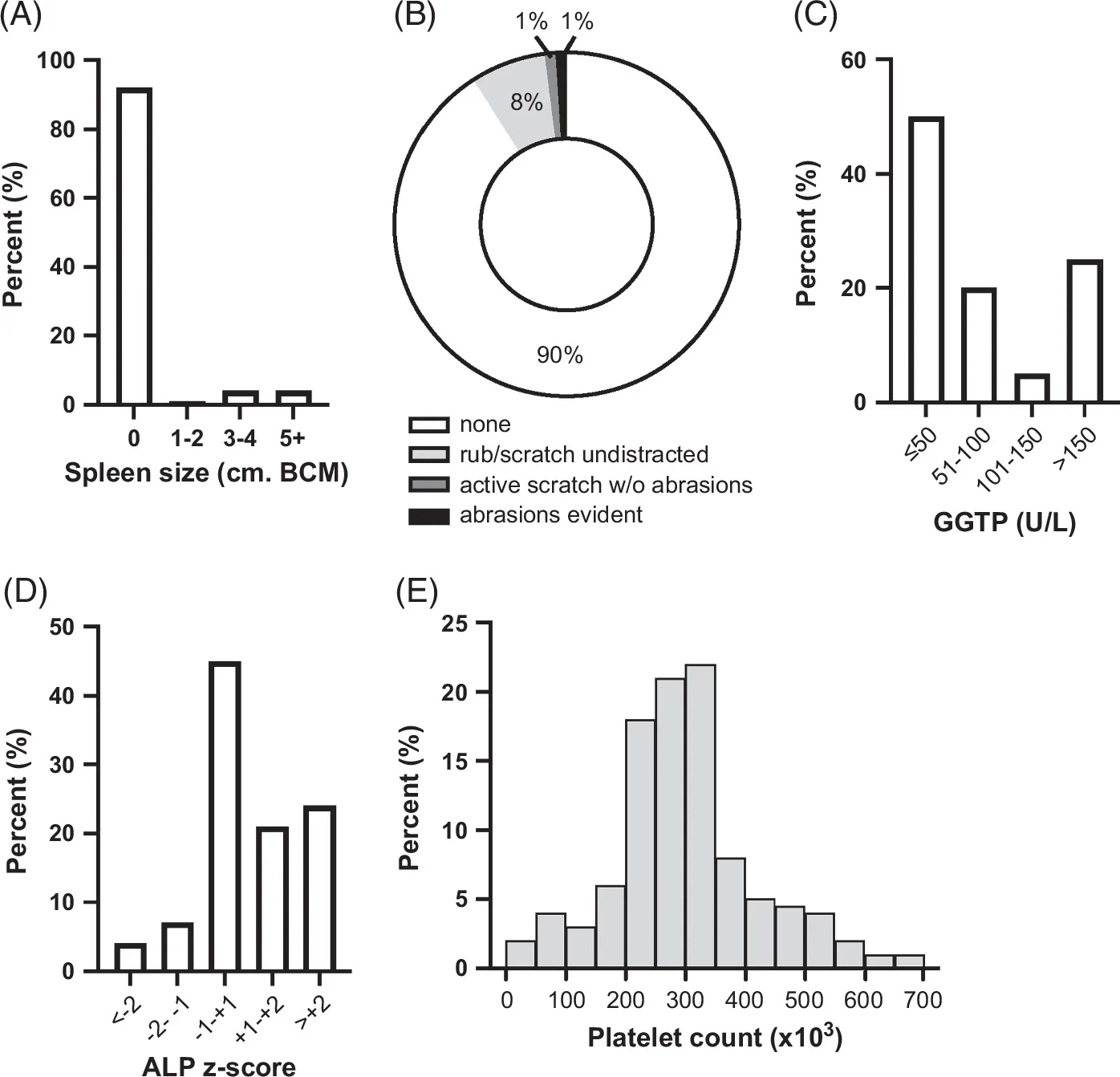
The severity of liver disease is mild in the majority of the pediatric PSC cohort at enrollment. Percentage frequency of (A) palpable splenomegaly [defined as centimeters below the costal margin (BCM)], (B) pruritus severity, (C) GGT levels, (D) ALP ULN z-score, and (E) platelet count levels. Abbreviations: ALP, alkaline phosphatase; BCM, below the costal margin; GGT, gamma-glutamyl transferase; PSC, primary sclerosing cholangitis; ULN, upper limit of normal.

Physical exam findings at enrollment revealed that the vast majority of participants had adequate growth; only 3% had weight-for-age z-scores of <−2, and 5% had height-for-age z-scores of <−2. Physical exam findings of portal hypertension included splenomegaly and ascites; 8% had a spleen palpable below the left costal margin (Figure 2A), and no participants had ascites. Pruritus with skin excoriations was assessed, and only 1% had evidence of skin abrasions from pruritus (Figure 2B).

Median laboratory values related to liver disease *at PSC diagnosis* were significantly higher compared with those obtained at study enrollment, with normal or mildly elevated median values of liver aminotransferases and GGT at enrollment for the entire cohort (Table 2). An additional analysis of a matched comparison between diagnosis and enrollment laboratory values in only those participants who had a specific lab value at both time points revealed similarly significant improvement at enrollment compared with diagnosis (Supplemental Table S4, https://links.lww.com/HC9/C488). It has been previously reported that a GGT of <50 U/L 1 year after diagnosis predicted good outcomes.^12^ The GGT at enrollment in the current study was <50 U/L in 50% of participants and >150 U/L in 25% (Figure 2C). Similarly, the ALP at enrollment was <1× ULN in 55% and >2× ULN in 24% (Figure 2D). With regard to laboratory evidence of portal hypertension and hypersplenism denoted by thrombocytopenia, the platelet count at enrollment was <150,000/μL in only 8%; the range of platelet counts is shown in Figure 2E.

**TABLE 2:** Laboratory values at PSC diagnosis and at enrollment

	At diagnosis		At enrollment		
	N	n (%) or median (range)	N	n (%) or median (range)	^a^ *p*
Total bilirubin (mg/dL)	146	0.6 (0.1, 11.6)	139	0.6 (0.2, 62.0)	0.956
AST (U/L)	154	71 (12, 1150)	152	36 (13, 733)	<0.001
ALT (U/L)	156	118 (10, 1150)	152	39 (8, 1460)	<0.001
ALP (U/L)	156	267 (65, 1772)	148	188 (33, 1226)	<0.001
Albumin (g/dL)	151	4.2 (2.3, 5.0)	138	4.3 (2.0, 5.4)	<0.001
Total protein (g/dL)	138	7.9 (0.8, 10.5)	122	7.6 (5.7, 10.4)	0.037
GGT (U/L)	142	204 (9, 1669)	137	53 (10, 1579)	<0.001
Hematocrit (%)	157	38 (21, 52)	146	41 (27, 51)	<0.001
WBC (×10^6^/μL)	153	8 (0, 26)	140	7 (0, 18)	<0.001
Platelets (×10^3^/μL)	155	339 (84, 1193)	138	290 (24, 652)	<0.001
APRI	150	0.6 (0.0, 11.8)	136	0.3 (0.1, 37.2)	<0.001
FIB-4	150	0.3 (0.0, 4.2)	135	0.3 (0.0, 16.3)	0.004

^a^ Signed rank test for difference between paired samples for participants with labs at both diagnosis and enrollment, adjusted for multiple comparisons using the adaptive step-up Bonferroni method.

Abbreviations: ALP, alkaline phosphatase; ALT, alanine transaminase; APRI, AST-to-platelet ratio index; AST, aspartate aminotransferase; FIB-4, Fibrosis-4 index; GGT, gamma-glutamyl transferase; PSC, primary sclerosing cholangitis.

**FIGURE 3:**
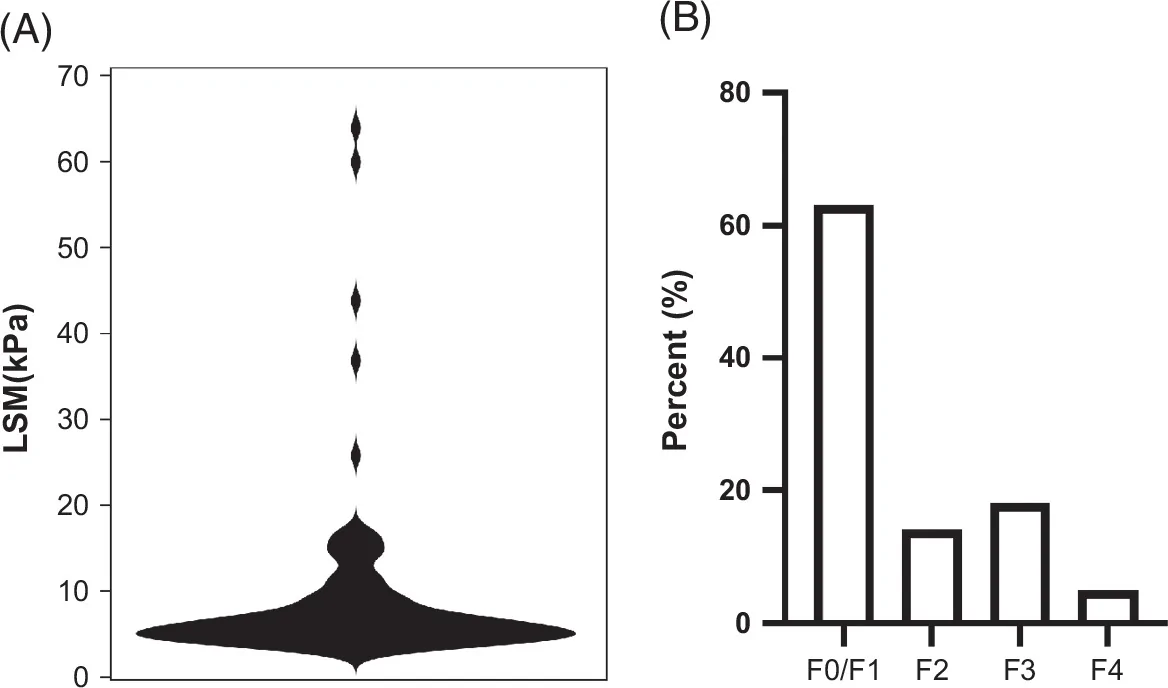
FibroScan LSM at enrollment. (A) Violin plot of LSM values. (B) Percentage frequency of predicted liver fibrosis stage based on LSM (F0/F1 ≤7 kPa; F2 7.1–9 kPa; F3 9.1–17 kPa; F4>17 kPa).^13^ Abbreviation: LSM, liver stiffness measurement.

Transient elastography (FibroScan) was performed on all participants at enrollment; however, only 66% were included in the final analyses due to the following: 17% had a lack of an associated clinical research form completed, 7% IQR >30%/probe size issue/<10 valid measurements, 6% not fasted/declined, and 4% other reasons. In all, 77% of participants within the analyzed FibroScan reports had fasted for ≥4 hours [median fasting time was 6 h (range 0–23)]. The median liver stiffness measurement (LSM) was 5.9 kPa (range 3–63.9); 63% of participants had a LSM of ≤7 kPa (fibrosis stage F0/F1), and 23% had LSM >9 kPa (fibrosis stage F3/4)^13^ (Figure 3). Comparison of laboratory values within the F0/F1 versus F3/F4 cohorts revealed significantly higher AST, ALT, ALP, GGT, IgG, APRI, and FIB-4 in the F3/F4 cohort (Table 3). There was no significant association of the use of ursodeoxycholic acid, vancomycin, or immunosuppression with LSM (Table 3).

**TABLE 3:** Baseline liver stiffness measurement (LSM) in relation to laboratory parameters and medication use

	LSM≤7 kPa (F0/F1) (N=72)		LSM>9 kPa (F3/F4) (N=27)		
	N	Median (range)	N	Median (range)	*p* ^a^
Total bilirubin (mg/dL)	64	0.5 (0.2, 1.7)	21	0.6 (0.3, 4.2)	0.150
AST (U/L)	68	31 (13, 727)	25	61 (23, 733)	<0.001
ALT (U/L)	69	29 (9, 816)	25	73 (15, 1460)	<0.001
ALP (U/L)	66	157 (51, 349)	25	227 (69, 1226)	0.024
GGT (U/L)	59	26 (11, 592)	23	168 (24, 1193)	<0.001
Total IgG (g/dL)	59	1211 (480, 2710)	25	1453 (534, 4050)	0.023
+ autoantibody enrollment	64	38 (59.4%)	22	12 (54.5%)	1.000
Platelets (×10^3^/μL)	63	296 (28, 558)	22	264 (51, 481)	1.000
APRI	62	0.3 (0.1, 6.0)	22	0.8 (0.1, 5.4)	<0.001
FIB-4	62	0.3 (0.0, 1.5)	22	0.5 (0.2, 3.8)	0.004
Ursodeoxycholic acid	72	29 (40.3%)	27	19 (70.4%)	0.074
Oral vancomycin	72	18 (25%)	27	7 (25.9%)	1.000
Any immunosuppressant	72	38 (52.8%)	27	16 (59.3%)	1.000
Any biologic therapy	72	25 (34.7%)	27	3 (11.1%)	0.144

^a^ The Fisher exact or Wilcoxon rank sum test, adjusted for multiple comparisons using the adaptive step-up Bonferroni method.

Abbreviations: ALP, alkaline phosphatase; ALT, alanine transaminase; APRI, AST-to-platelet ratio index; AST, aspartate aminotransferase; FIB-4, Fibrosis-4 index; GGT, gamma-glutamyl transferase; IgG, immunoglobulin G; PSC, primary sclerosing cholangitis.

### Characterization of IBD in the pediatric PSC cohort

**FIGURE 4:**
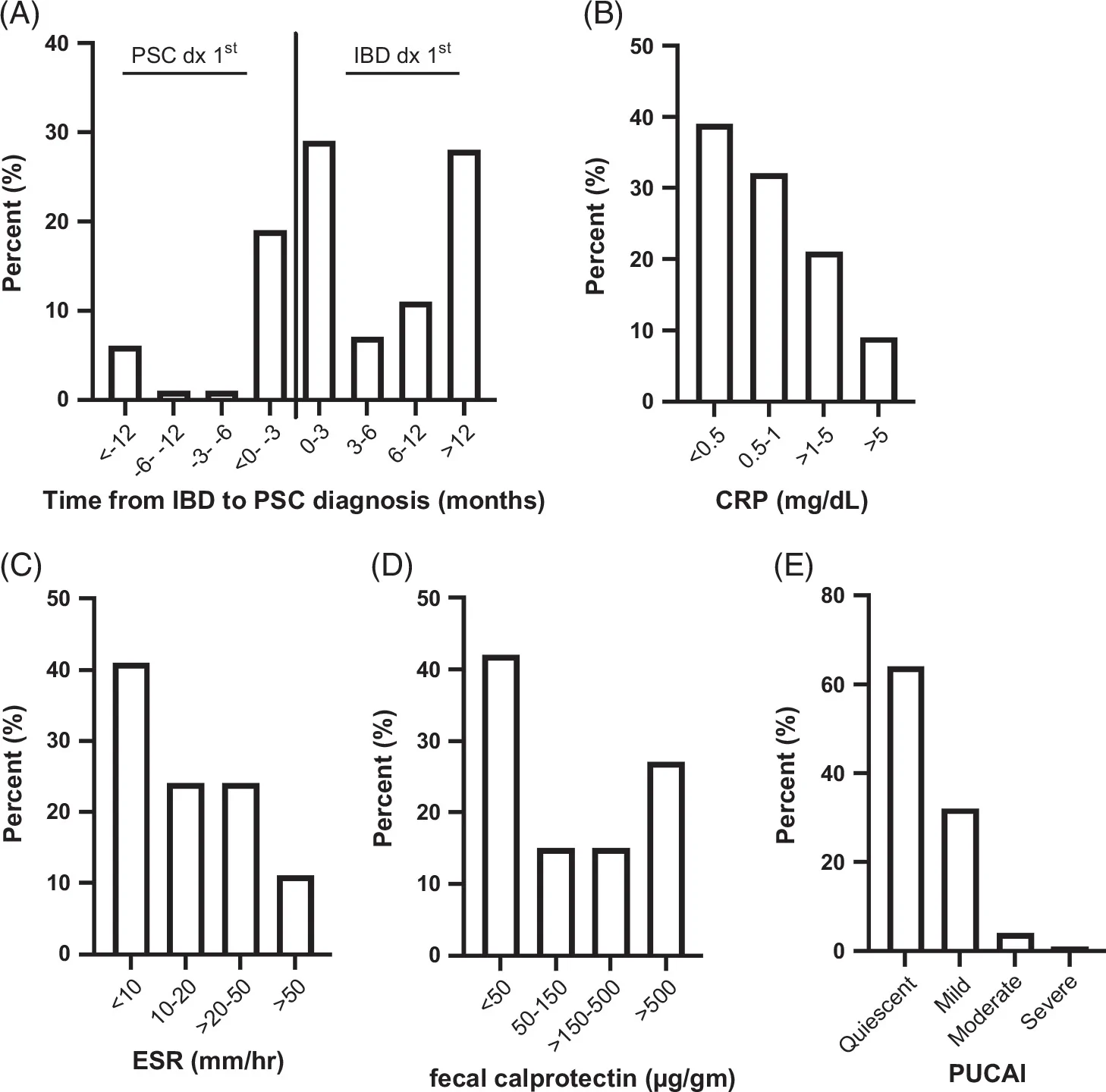
Characterization of IBD in the pediatric PSC cohort at enrollment. Percentage frequency of (A) time from IBD to PSC diagnosis (months), (B) CRP (mg/dL), (C) ESR (mm/h), (D) fecal calprotectin (μg/g), and (E) PUCAI score. Abbreviations: CRP, C-reactive protein; ESR, erythrocyte sedimentation rate; IBD, inflammatory bowel disease; PSC, primary sclerosing cholangitis; PUCAI, pediatric ulcerative colitis activity index.

As expected, the majority of participants had IBD at study enrollment (74%). Ulcerative colitis (UC) was most prevalent (67%), followed by 22% with Crohn disease and 11% as unclassified or indeterminate. The median duration of IBD before enrollment was 3.6 years (range 0–15.9). The median duration of IBD before the diagnosis of PSC was 2.4 months (range: 31–149.7). In 55% of participants with IBD, the diagnosis of PSC occurred within 0–3 months of the IBD diagnosis. Interestingly, 26% of the cohort were diagnosed with PSC before the IBD diagnosis, and 28% were diagnosed with PSC ≥1 year after the IBD diagnosis (Figure 4A).

The severity of IBD *at the time of IBD diagnosis* was characterized by multimodal assessments, including the Physician Global Assessment (PGA) and endoscopic and biochemical parameters (Supplemental Table S5, https://links.lww.com/HC9/C488). Based on the PGA, 44% of UC participants had quiescent/mild disease, 48% had moderate disease, and 7% had severe UC. Of the participants with Crohn disease, 60% were inactive/mild disease, and 40% had moderate/severe disease. Endoscopically, extensive colonic disease was prominent, with similar frequencies of macroscopic involvement from the cecum to the left colon (81%–86%); rectal involvement was slightly less prevalent (74%). The severity of macroscopic findings at the diagnostic colonoscopy revealed that 52% had no visible/mild inflammation and 48% had moderate/severe inflammation. The right colon was more severely inflamed compared with the left colon in 25%, and rectal sparing occurred in 25%. Backwash ileitis was described in 14%, and microscopic ileitis in 45%. There were no reports of perianal disease. Eight participants (5%) had had a colectomy, and 63% reported post-colectomy pouchitis.

The severity of IBD at study enrollment was characterized based on the pediatric ulcerative colitis activity index (PUCAI) score that was utilized for both UC and Crohn disease participants, as well as biochemical parameters. The PUCAI scores at enrollment showed that 64% had quiescent disease (PUCAI score <10), 32% had mild disease (score 10–34), and 5% had moderate (score 35–64)/severe (score >64) disease. Similarly, serologies revealed minimal evidence of intestinal inflammation: median C-reactive protein (CRP) 1 mg/dL (range 0–14; N=44), median erythrocyte sedimentation rate (ESR) 14 mm/h (range 0–74; N=71), and ~10% of participants had high CRP (>5 mg/dL) and ESR (>50 mm/h). Interestingly, the fecal calprotectin showed evidence of moderate inflammation, with 27% of participants displaying a fecal calprotectin of >500 μg/g (Figures 4B–E). One participant had been diagnosed with colorectal carcinoma at 14 years of age.

### Characterization of AIH in the pediatric PSC cohort

**FIGURE 5:**
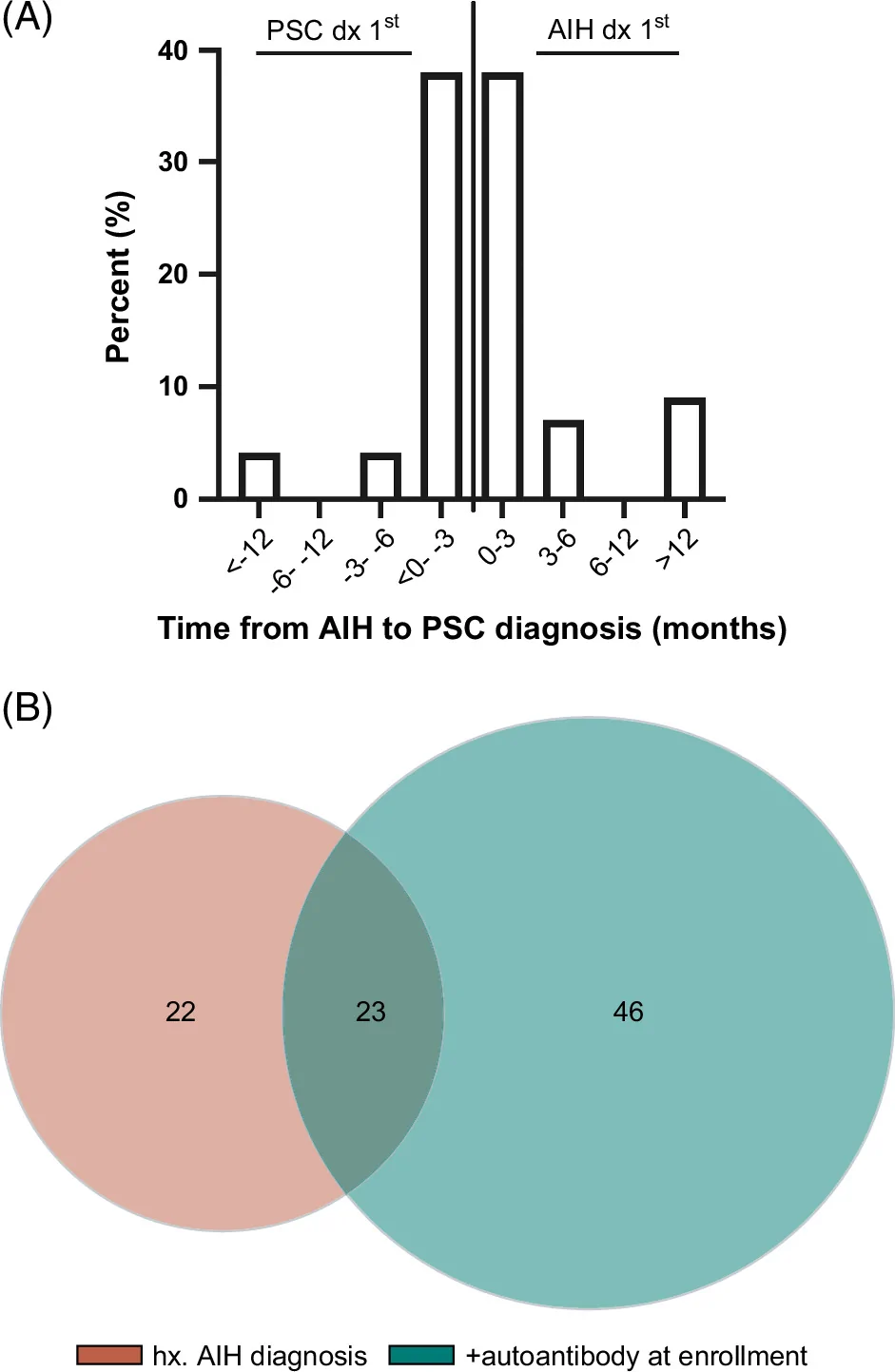
Characterization of autoantibody positivity in the pediatric PSC cohort. (A) Percentage frequency of time from AIH to PSC diagnosis (months). (B) Venn diagram of participants positive for at least one autoantibody at enrollment (N=69; green) and participants with a previous diagnosis of AIH (N=45; pink). Abbreviations: AIH, autoimmune hepatitis; PSC, primary sclerosing cholangitis.

Within the entire PSC cohort (175 participants), a diagnosis of AIH was reported in 26% (Figure 1B). Of the 131 participants who had data related to the *diagnostic workup of AIH*, 34% were given a diagnosis of AIH based on the presence of autoantibodies, elevated IgG, and characteristic histologic findings of AIH (ie, 100% of participants had the presence of lymphocytic interface hepatitis). The diagnosis of PSC preceded the diagnosis of AIH in 47% of the cohort. AIH was diagnosed within 0–3 months of the diagnosis of PSC in 38%, and 9% of participants were diagnosed with AIH ≥1 year after the PSC diagnosis (Figure 5A).

### High frequency of autoantibody positivity at enrollment in the absence of previous AIH diagnosis

The autoantibody profile at the *time of AIH diagnosis* (45 participants) included 57% ANA-positive, 55% anti-actin/ASMA-positive, and 6% LKM antibody–positive. The median IgG level at the time of AIH diagnosis was elevated at 1910 mg/dL (range 922–4760) and decreased to a median of 1552 mg/dL (range 902–3329) at enrollment for this cohort. At enrollment, autoantibodies and total IgG were measured in all participants, including those without a previous diagnosis of AIH. The median total IgG level at enrollment for the entire cohort was 1324 mg/dL (range 480–5171). Of the 118 participants tested for ANA, 48% were positive; of the 82 tested for anti-actin/ ASMA, 34% were positive; and of the 109 tested for LKM, 11% were positive (Supplemental Table S6, https://links.lww.com/HC9/C488). A variety of autoantibody assays were used, making direct comparisons of antibody levels unreliable. The frequency of being positive for at least one autoantibody at enrollment was 56% (69 of 124 participants). Comparison of the 69 participants with a positive autoantibody at enrollment with the 45 participants who were previously diagnosed with AIH before enrollment revealed that 67% (N=46) with a positive autoantibody at enrollment were outside of the cohort of those with AIH (Figure 5B). With regard to the 45 participants with a previous diagnosis of AIH, 23 (50%) remained autoantibody-positive at enrollment, and 67% were on an immunosuppressant at enrollment (see list of immunosuppressants/anti-inflammatories within Supplemental Table S1, https://links.lww.com/HC9/C488). Of the previously diagnosed AIH cohort, there was no significant difference in the percent with use of an immunosuppressant between those who were autoantibody-positive (70%) versus autoantibody-negative (55%) at enrollment (*p*=0.4).

### Limited unique phenotypic characteristics related to small-duct-only PSC or to PSC with or without IBD and/or AIH

Analysis of various phenotypes was performed to determine if there were characteristics that were unique to a given phenotype. Characteristics at enrollment that were assessed included demographics, duration of PSC, laboratory values including autoantibodies, biomarkers of fibrosis (APRI, FIB-4, LSM), and medications. Comparison of the following phenotypes included: large-duct versus small-duct-only PSC (Supplemental Table S7A, https://links.lww.com/HC9/C488), enrollment autoantibody-positive versus autoantibody-negative PSC (Supplemental Table S7B, https://links.lww.com/HC9/C488), PSC with or without IBD (Supplemental Table S7C, https://links.lww.com/HC9/C488), and PSC with/without IBD and/or AIH (Supplemental Table S7D, https://links.lww.com/HC9/C488). There were no significant unique characteristics between large and small-duct-only PSC (Supplemental Table S7A, https://links.lww.com/HC9/C488). Comparison of the autoantibody-positive versus autoantibody-negative PSC cohorts (Supplemental Table S7B, https://links.lww.com/HC9/C488) revealed that 83% of the autoantibody-positive cohort had IBD compared with 60% of the autoantibody-negative cohort (*p*=0.119). Comparison of with/without IBD revealed an expected significantly increased use of biologics in the IBD cohorts (Supplemental Table S7C, D, https://links.lww.com/HC9/C488). In addition, there was a higher frequency of autoantibody positivity in the PSC/IBD cohort (63%) compared with PSC without IBD (35%) (*p*=0.127; Supplemental Table S7C, https://links.lww.com/HC9/C488) and in the PSC/IBD/AIH cohort (78%), compared with PSC only (30%), PSC/IBD (58%), and PSC/AIH (46%) (*p*=0.067; Supplemental Table S7D, https://links.lww.com/HC9/C488). Furthermore, the PSC/AIH cohort had significantly higher use of ursodeoxycholic acid (86%) compared with other groups (40%–65%; *p*=0.021), and there was a higher median LSM in the PSC/AIH cohort (10.7 kPa) compared with other groups (LSMs of 5.8 kPa; *p*=0.079; Supplemental Table S7D).

## DISCUSSION

This study represents the characterization of a large prospectively enrolled cohort of children with PSC who were extensively phenotyped using detailed demographic, clinical, biochemical, histologic, and radiologic data. Despite the ascertainment of this cohort at tertiary referral liver centers in North America, the majority of participants had mild liver disease with a median total bilirubin in the cohort of 0.6 mg/dL, and only 8% having evidence of possible portal hypertension. This was supported by transient elastography data, which demonstrated that over 60% of participants had an LSM of ≤7 kPa (fibrosis stage F0/F1) and only 5% had an LSM>17 kPa (fibrosis stage F4). Incorporating clinical and laboratory data together within the SCOPE index score obtained at diagnosis revealed that only 12% had a high risk of progression of disease within 5 years of the original diagnosis. The fact that the majority of the current cohort are established (prevalent) cases of PSC could influence the severity of disease, based on selection/survivorship bias, referral patterns, and treatment effects from ursodiol and/ or vancomycin. This picture of mild liver disease in our pediatric PSC cohort contrasts with a previous large, retrospective international cohort study in which, after 10 years of disease, 38% of patients had portal hypertension and 25% had biliary complications. Once complications developed, the median survival with native liver was 2.8 years for those with portal hypertension and 3.5 years with biliary complications.^6^

The current study also revealed that the SCOPE index and the FibroScan LSM correlated with elevated biochemistries, suggesting that they may serve as potential biomarkers of disease severity. If one assumes that LSM is a good indication of fibrosis in PSC, and there is a relationship between LSM and liver biochemistries, then perhaps liver biochemistries (APRI, FIB-4) are also a reliable tool to monitor PSC progression. It should be noted that the limitations related to LSM in this study include that only two-thirds of participants underwent FibroScan, and of those, 23% were not fasting. Furthermore, the predicted fibrosis stage cutoffs were extrapolated from adult data, as no validated pediatric cutoffs exist. Based on our cross-sectional evidence supportive of the use of FibroScan as a biomarker of disease progression and fibrosis severity, a future longitudinal validation study is ongoing. Future directions could also include evaluation of APRI, FIB-4, ALT, and GGT thresholds associated with LSM (AUROC analyses).

The mild disease severity of the current cohort is also reflected by the low GGT, which was <50 U/L in 50% of participants. Normalization of GGT has previously been noted to reflect a favorable 5-year outcome in patients with PSC.^12^,^14^ It might be speculated that this normal GGT reflects that the majority of our study cohort were receiving either ursodiol (51%) and/or vancomycin (26%). However, a large retrospective case-control study of PSC patients matched for treatment with ursodiol, vancomycin, or observation only found no discernible difference in outcomes in terms of GGT normalization or liver fibrosis, regardless of treatment modality.^15^ Rather, GGT normalization for any reason was associated with a favorable prognosis. It will be important for the current longitudinal study to determine prospectively over the coming years if GGT normalization is similarly predictive of better clinical outcomes and is unrelated to medication usage.

As expected, 74% of patients in this study had IBD at enrollment, with 96% displaying quiescent or mild IBD based on PUCAI and serologic markers of inflammation (ESR, CRP). In contrast, 27% of the cohort had high fecal calprotectin levels (>500 μg/g), reinforcing the concept of “subclinical gut inflammation” in IBD and the need for objective markers of gut inflammation such as fecal calprotectin and/or endoscopy.^16^ Given the high prevalence of concurrent IBD and PSC, patients diagnosed first with PSC should be screened with fecal calprotectin for asymptomatic IBD annually.^17^ Recently, Ricciuto et al^18^ demonstrated that PSC/IBD patients on oral vancomycin for a minimum of 3 months had higher IBD remission rates than those not treated with oral vancomycin. As such, it is possible that 26% of the cohort being treated with oral vancomycin may be contributing to the mild IBD observed. In addition, the common use of biologics in pediatric IBD patients in recent years may have contributed to the milder IBD phenotype in the current study cohorts. While cholangiocarcinoma was not reported in this cohort, and colorectal cancer was reported in only 1 patient, the risk for development of these malignancies remains a valid concern and requires ongoing clinical screening as well.^19^ A retrospective study noted that pediatric patients with IBD and PSC had a 28-fold higher risk of cancer than those in the general population.^20^

A notable feature of this cohort was the high rate of autoantibody positivity. More than half of the participants were positive for at least one autoantibody at enrollment. Interestingly, 45% were positive for ANA, and 34% were positive for anti-actin/anti-SMA; autoantibodies that are traditionally considered sensitive biomarkers for AIH.^21^,^22^ The majority of participants with a positive autoantibody at enrollment were outside of the cohort of those diagnosed with AIH before enrollment, suggesting that autoantibodies do not necessarily reflect classical AIH in children with PSC. In fact, the highest frequency of autoantibodies occurred in the cohorts with IBD, suggesting that the antibodies are related to IBD, not AIH or PSC. ANA positivity has been reported in IBD patients treated with anti-tumor necrosis factor therapies,^23^ and decades ago, anti-actin and anti-SMA were reported frequently within IBD patients.^24^,^25^ This finding reinforces the necessity for liver histology before assigning a label of overlap syndrome.

We did not find a specific clinical phenotype based on comparisons of large-duct versus small-duct-only PSC, or concurrent IBD and/or AIH. Conversely, it has previously been shown that concurrent IBD^26^ or small-duct-only PSC^27^ have less severe disease than other phenotypes. In our study, there was a trend toward a more severe phenotype within the PSC/AIH cohort. This phenotype had the highest frequency of focal strictures on diagnostic MRCP and the highest incidence of periductal inflammation and injury on liver histology. Finally, the PSC/AIH phenotype displayed a higher median LSM at the time of study enrollment compared with other phenotypes. These differences may be related to the overlay of AIH on PSC for undetermined lengths of time. However, there has been a recent paradigm shift in the classification of PSC/AIH as a distinct entity.^28^ Some groups have suggested that the AIH phenotype that concomitantly exists in some pediatric PSC patients actually represents an inflammatory phase of the disease, which inevitably evolves to PSC alone. With this concept in mind, it may be difficult to make clear comparisons between PSC/AIH and PSC alone if these are actually different phases of the same disease process.

The study has several limitations. The majority of the participants were prevalent participants with different durations of disease at study enrollment. Secondly, medications used to treat PSC were driven by individual center protocols, thereby eliminating the ability to understand the impact of usage on clinical outcomes. Thirdly, with regard to missingness of data, missing data occurred roughly in equal proportions in each category in the presented tables, suggesting there is no systematic pattern to the missingness. It should be noted that some data elements in this study are obtained from clinical records and are thus subject to local clinical practice and possibly reimbursement restrictions. This may be the case in Supplemental Table S3, https://links.lww.com/HC9/C488, where more observations are missing in the Low Risk SCOPE Index group than in the others. Clinicians may be less likely to order labs for patients assessed to be low risk. In other cases, such as with Supplemental Table S7A, https://links.lww.com/HC9/C488, a sensitivity analysis suggests that information added by missing data would be unlikely to meaningfully change the presented results. We do not believe these results are affected by the observed patterns of missing data.

In summary, this prospective cohort of children with PSC revealed mild liver and intestinal disease in the majority. Laboratory biomarkers of liver injury and fibrosis were associated with LSM severity, suggesting LSM as a reliable tool to monitor pediatric PSC progression. This dataset encompasses a well-phenotyped cohort of children with PSC, which serves as a foundation for ongoing prospective data collection to define the contemporary natural history of pediatric PSC and its predictors of outcomes. The changing landscape of IBD therapies for pediatric patients may also alter the natural history of pediatric PSC-IBD. In the future, well-powered prospective clinical trials in patients with PSC and concurrent AIH and/or IBD may allow critical assessment of biomarkers and the impact of therapeutic medications on disease progression.

## Supplementary Material
