# Supplementary material for: The Childhood Liver Disease Research Network's prospective characterization of pediatric primary sclerosing cholangitis
Source: Hepatol Commun. 2026 Sep 18;10(10):e1049. doi: 10.1097/HC9.0000000000001049 (PMC13592951; doi:10.1097/HC9.0000000000001049)
Supplement: Supplementary file 1 [file hc9-10-e1049-s002.docx]

**SDC Table 1. Medications at Enrollment**

| **Medication** | **N** | **n (%)** |
| --- | --- | --- |
| **Anti-pruritic therapy** | | |
| Ursodeoxycholic acid | 164 | 84 (51.2%) |
| Cholestyramine | 160 | 2 (1.3%) |
| Naltrexone | 160 | 1 (0.6%) |
| Rifampin | 160 | 4 (2.5%) |
| Sertraline | 160 | 3 (1.9%) |
| **Antibiotics & Probiotics** | | |
| Oral Vancomycin | 164 | 44 (26.8%) |
| Ciprofloxacin | 161 | 5 (3.1%) |
| Metronidazole | 161 | 2 (1.2%) |
| Trimethoprim / Sulfamethoxazole | 161 | 1 (0.6%) |
| Amoxicillin | 161 | 1 (0.6%) |
| Other antibiotic | 161 | 7 (4.3%) |
| Probiotic therapy | 161 | 10 (6.2%) |
| **Immunosuppression / Anti-inflammatory Therapy** | | |
| Any Immunosuppressant | 164 | 87 (53%) |
| Prednisone / prednisolone | 164 | 22 (13.4%) |
| Budesonide | 164 | 13 (7.9%) |
| Methylprednisolone | 164 | 4 (2.4%) |
| Hydrocortisone | 164 | 3 (1.8%) |
| Azathioprine | 164 | 29 (17.7%) |
| Mycophenolate mofetil | 164 | 6 (3.7%) |
| Tacrolimus | 164 | 3 (1.8%) |
| Methotrexate | 164 | 1 (0.6%) |
| Aminosalicylate / Aminosalicylic acid | 164 | 26 (15.9%) |
| **Immunobiological therapy** | | |
| Infliximab | 164 | 26 (15.9%) |
| Adalimumab | 164 | 9 (5.5%) |
| Vedolizimab | 164 | 16 (9.8%) |
| Ustekinumab | 164 | 8 (4.9%) |
| **Diuretics** | | |
| Furosemide | 164 | 1 (0.6%) |
| Spironolactone | 164 | 3 (1.8%) |
| **Vitamins** | | |
| DEKA Multivitamins | 164 | 16 (9.8%) |
| Vitamin A | 164 | 1 (0.6%) |
| Vitamin D | 164 | 73 (44.5%) |
| Vitamin E | 164 | 4 (2.4%) |
| Vitamin K | 164 | 3 (1.8%) |

Medications not reported in this cohort: Colesevelam, Neurontin, Augmentin, 6-Mercaptopurine, Cyclosporine, Rapamycin, Tofacitinib, Certolizumab, Golimumab, Natalizumab

**SDC Table 2. Diagnostic MCRP & Histologic Features of PSC by Phenotype**

|  | **PSC only (N=37)** | | **PSC/IBD (N=93)** | | **PSC/AIH (N=14)** | | **PSC/IBD/AIH (N=31)** | |  |
| --- | --- | --- | --- | --- | --- | --- | --- | --- | --- |
| **Feature** | **N** | **n (%)** | **N** | **n (%)** | **N** | **n (%)** | **N** | **n (%)** | **P-value*** |
| Large Duct PSC | | | | | | | | | |
| Focal stricturing of bile duct(s) | 31 | 19 (61.3%) | 77 | 53 (68.8%) | 11 | 11 (100%) | 22 | 12 (54.5%) | 0.074 |
| Dominant stricture of the common bile duct | 31 | 7 (22.6%) | 77 | 16 (20.8%) | 11 | 4 (36.4%) | 22 | 7 (31.8%) | 0.615 |
| Saccular dilatation of bile duct(s) | 31 | 2 (6.5%) | 77 | 8 (10.4%) | 11 | 2 (18.2%) | 22 | 3 (13.6%) | 0.615 |
| Beaded appearance of bile duct(s) | 31 | 21 (67.7%) | 77 | 45 (58.4%) | 11 | 5 (45.5%) | 22 | 15 (68.2%) | 0.615 |
| Pruning appearance of the distal bile duct branches | 31 | 8 (25.8%) | 77 | 29 (37.7%) | 11 | 3 (27.3%) | 22 | 11 (50%) | 0.615 |
| Small Duct Only PSC | | | | | | | | | |
| Periductal edema | 13 | 8 (61.5%) | 22 | 8 (36.4%) | 3 | 2 (66.7%) | 12 | 4 (33.3%) | 0.615 |
| Concentric inflammation | 13 | 4 (30.8%) | 22 | 9 (40.9%) | 3 | 3 (100%) | 12 | 6 (50%) | 0.397 |
| Bile duct injury | 13 | 9 (69.2%) | 22 | 12 (54.5%) | 3 | 3 (100%) | 12 | 11 (91.7%) | 0.210 |
| Ductular reaction | 13 | 9 (69.2%) | 22 | 11 (50%) | 3 | 2 (66.7%) | 12 | 11 (91.7%) | 0.174 |
| Neutrophils in bile ducts (cholangitis) | 13 | 8 (61.5%) | 22 | 7 (31.8%) | 3 | 3 (100%) | 12 | 7 (58.3%) | 0.138 |
| Periductal fibrosis/'onion skinning' around interlobular bile ducts or smaller profiles | 13 | 8 (61.5%) | 22 | 16 (72.7%) | 3 | 1 (33.3%) | 12 | 9 (75%) | 0.615 |

* Fisher’s exact test, adjusted for multiple comparisons using the adaptive step-up Bonferroni method.

**SDC Table 3. Association of SCOPE Index^#^ at Diagnosis with Labs at Enrollment**

|  | **Low risk (0-3) (N=51)** | | **Medium risk (4-5) (N=59)** | | **High risk (6-11) (N=16)** | |  |
| --- | --- | --- | --- | --- | --- | --- | --- |
| **Enrollment labs** | **N** | **median (range)** | **N** | **median (range)** | **N** | **median (range)** | **P-value*** |
| Total bilirubin (mg/dL) | 40 | 0.5 (0.2, 3.6) | 53 | 0.6 (0.2, 3.1) | 14 | 1.0 (0.3, 62.0) | 0.001 |
| AST (U/L) | 45 | 37 (13, 727) | 57 | 33 (13, 733) | 14 | 86 (16, 677) | 0.002 |
| ALT (U/L) | 44 | 34 (8, 816) | 58 | 35 (10, 756) | 14 | 76 (9, 1460) | 0. 002 |
| ALP (U/L) | 43 | 153 (51, 693) | 55 | 192 (52, 1226) | 14 | 293 (33, 1005) | 0.227 |
| Albumin (g/dL) | 40 | 4.5 (3.3, 5.0) | 53 | 4.3 (2.0, 5.3) | 13 | 3.9 (3.4, 5.1) | 0.052 |
| Total Protein (g/dL) | 37 | 7.8 (6.9, 8.7) | 45 | 7.6 (5.7, 10.0) | 13 | 8.0 (6.5, 9.4) | 0.142 |
| GGTP (U/L) | 39 | 30 (11, 870) | 56 | 55 (12, 723) | 11 | 163 (18, 1579) | 0.005 |
| Hematocrit (%) | 44 | 43 (32, 51) | 55 | 41 (30, 50) | 14 | 41 (27, 48) | 0.072 |
| WBC (x10^6^/μl) | 42 | 6 (0, 12) | 55 | 7 (3, 18) | 13 | 7 (3, 12) | 0.323 |
| Platelet count (x10^3^/μl) | 42 | 286 (162, 537) | 52 | 305 (28, 556) | 13 | 248 (24, 652) | 0.040 |
| APRI | 42 | 0.3 (0.1, 6.0) | 51 | 0.3 (0.1, 5.4) | 13 | 1.8 (0.1, 37.2) | <0.001 |
| FIB-4 | 41 | 0.3 (0.0, 1.5) | 51 | 0.3 (0.1, 3.8) | 13 | 0.5 (0.2, 16.3) | 0.008 |

^#^Sclerosing Cholangitis Outcomes in PEdiatrics (SCOPE) Index: prognostic and risk-stratification for poor outcome (low, medium, high)^10^

* From linear regression model log(lab) ~ SCOPE adjusted for duration of PSC; all p-values adjusted for multiple comparisons using the adaptive step-up Bonferroni method.

**SDC Table 4. Laboratory Values at PSC Diagnosis and at Enrollment: A Paired Comparison**

|  | **At diagnosis** | | **At enrollment** | |  |
| --- | --- | --- | --- | --- | --- |
|  | **N** | **n (%) or median (range)** | **N** | **n (%) or median (range)** | **P-value*** |
| Total bilirubin (mg/dL) | 124 | 0.6 (0.1, 11.6) | 124 | 0.6 (0.2, 62.0) | 0.956 |
| AST (U/L) | 141 | 70 (12, 1150) | 141 | 36 (13, 733) | <.001 |
| ALT (U/L) | 143 | 118 (10, 1150) | 143 | 37 (8, 1460) | <.001 |
| Alkaline phosphatase (U/L) | 139 | 264 (65, 1772) | 139 | 189 (33, 1226) | <.001 |
| Albumin (g/dL) | 126 | 4.2 (2.3, 5.0) | 126 | 4.3 (2.0, 5.3) | <.001 |
| Total Protein (g/dL) | 110 | 7.9 (2.0, 10.3) | 110 | 7.6 (5.7, 10.0) | 0.037 |
| GGTP (U/L) | 119 | 210 (14, 1669) | 119 | 49 (11, 1579) | <.001 |
| Hematocrit (%) | 139 | 38 (21, 52) | 139 | 41 (27, 51) | <.001 |
| WBC | 132 | 8 (0, 26) | 132 | 7 (0, 18) | <.001 |
| Platelet count | 131 | 343 (84, 1193) | 131 | 295 (24, 652) | <.001 |
| APRI | 126 | 0.6 (0.0, 11.8) | 126 | 0.3 (0.1, 37.2) | <.001 |
| FIB-4 | 125 | 0.2 (0.0, 4.2) | 125 | 0.3 (0.0, 16.3) | 0.004 |

*Signed rank test for difference between paired samples, adjusted for multiple comparisons using

the adaptive step-up Bonferroni method.

**SDC Table 5. IBD Severity at IBD Diagnosis**

|  | | **Total N** | **n (%)** |
| --- | --- | --- | --- |
| **Physician global assessment (PGA) of IBD activity at IBD diagnosis** | | | |
| UC/IBD-U | None/Quiescent | 81 | 12 (14.8%) |
|  | Mild |  | 24 (29.6%) |
|  | Moderate |  | 39 (48.1%) |
|  | Severe |  | 6 (7.4%) |
| Crohn's disease | None/Quiescent | 30 | 6 (20%) |
|  | Mild |  | 12 (40%) |
|  | Moderate/Severe |  | 12 (40%) |
| **Location of macroscopic involvement at diagnosis** | | | |
| Esophagus |  | 107 | 12 (11.2%) |
| Stomach |  | 108 | 37 (34.3%) |
| Duodenum |  | 106 | 20 (18.9%) |
| Mid small bowel (jejunum/proximal ileum) |  | 47 | 5 (10.6%) |
| Terminal ileum |  | 103 | 30 (29.1%) |
| Cecum |  | 96 | 78 (81.3%) |
| Ascending colon |  | 110 | 92 (83.6%) |
| Transverse colon |  | 111 | 91 (82%) |
| Left colon (descending colon and/or sigmoid) |  | 110 | 95 (86.4%) |
| Rectum |  | 104 | 77 (74%) |
| **Features at diagnostic colonoscopy** | | | |
| Global endoscopic severity at diagnostic colonoscopy | No visible inflammation (macroscopically normal colonoscopy) | 116 | 10 (8.6%) |
|  | Mild inflammation (e.g., erythema, decreased vascular pattern, mild friability, edema) |  | 50 (43.1%) |
|  | Moderate/severe inflammation (e.g., ulcers, significant friability or spontaneous bleeding) |  | 56 (48.3%) |
| Right colon more severely inflamed than left colon |  | 85 | 21 (24.7%) |
| Rectal sparing |  | 87 | 22 (25.3%) |
| Backwash ileitis |  | 90 | 13 (14.4%) |
| Microscopic ileitis |  | 93 | 42 (45.2%) |
| Perianal disease (fistula, abscess) |  | 97 | 0 |
| **Colectomy** | | | |
| Colectomy (prior to enrollment) |  | 168 | 8 (4.8%) |
| Pouchitis |  | 8 | 5 (62.5%) |

**SDC Table 6. Positive Autoantibodies at Enrollment**

| **Autoantibody*** | | **N** | **n (%) or median (range)** |
| --- | --- | --- | --- |
| **ANA (antinuclear antibody test) Titer (N=118 tested)** | 1:2560 | 56 | 3 (5.4%) |
|  | 1:1280 |  | 7 (12.5%) |
|  | >=1:1280 |  | 2 (3.6%) |
|  | 1:640 |  | 5 (8.9%) |
|  | >1:640 |  | 1 (1.8%) |
|  | 1:320 |  | 14 (25%) |
|  | 1:160 |  | 12 (21.4%) |
|  | 1:80 |  | 11 (19.6%) |
|  | Positive |  | 1 (1.8%) |
| **ASMA (anti-actin) (N=82 tested)** | 27 units | 28 | 1 (3.6%) |
|  | 30 units |  | 1 (3.6%) |
|  | 36 units |  | 1 (3.6%) |
|  | 45 units |  | 1 (3.6%) |
|  | 106 units |  | 1 (3.6%) |
|  | >1:2560 |  | 1 (3.6%) |
|  | 1:320 |  | 1 (3.6%) |
|  | 1:160 |  | 2 (7.1%) |
|  | 1:80 |  | 6 (21.4%) |
|  | 1:40 |  | 4 (14.3%) |
|  | 1:20 |  | 7 (25%) |
|  | Neg/Pos 1+-4+: 1 |  | 1 (3.6%) |
|  | Neg/Pos 1+-4+: 2 |  | 1 (3.6%) |
| **Anti-LKM (N=109 tested)** | 20 units | 12 | 2 (2.2%) |
|  | 1:120 |  | 1 (1.1%) |
|  | 1:40 |  | 1 (1.1%) |
|  | 1:20 |  | 8 (9%) |

*The specific assay used to quantify a given antibody (i.e. titers versus ELISA quantification)
was based on the clinical assay used at the institution’s site.

**SDC Table 7A. Comparison of Large Duct vs. Small Duct Only PSC**

|  | | **Large Duct PSC (N=136)** | | **Small Duct Only PSC (N=39)** | |  |
| --- | --- | --- | --- | --- | --- | --- |
| **Variable** |  | **N** | **n (%) or median (range)** | **N** | **n (%) or median (range)** | **P-value*** |
| AIH at enrollment |  | 94 | 31 (33%) | 37 | 14 (37.8%) | 0.996 |
| IBD at enrollment |  | 129 | 96 (74.4%) | 39 | 28 (71.8%) | 0.996 |
| Age at consent (years) |  | 136 | 16.2 (4.2, 25.6) | 39 | 13.7 (2.5, 20.8) | 0.249 |
| Sex | Female | 133 | 57 (42.9%) | 39 | 12 (30.8%) | 0.996 |
|  | Male |  | 76 (57.1%) |  | 27 (69.2%) |  |
| Ethnicity | Hispanic | 123 | 8 (6.5%) | 34 | 4 (11.8%) | 0.996 |
|  | Non-Hispanic |  | 115 (93.5%) |  | 30 (88.2%) |  |
| Race | Asian | 125 | 6 (4.8%) | 35 | 0 (0%) | 0.996 |
|  | Black or African American |  | 10 (8%) |  | 1 (2.9%) |  |
|  | Multiracial |  | 1 (0.8%) |  | 1 (2.9%) |  |
|  | Native American/Pacific Islander |  | 0 (0%) |  | 1 (2.9%) |  |
|  | White |  | 108 (86.4%) |  | 32 (91.4%) |  |
| Length of PSC diagnosis (years) |  | 130 | 2.3 (0.1, 14.5) | 38 | 2.2 (0.1, 10.0) | 0.996 |
| Total bilirubin (mg/dL) |  | 107 | 0.6 (0.2, 62.0) | 32 | 0.6 (0.3, 2.0) | 0.996 |
| AST (U/L) |  | 116 | 38 (13, 733) | 36 | 35 (15, 152) | 0.996 |
| ALT (U/L) |  | 116 | 42 (9, 1460) | 36 | 32 (8, 448) | 0.996 |
| ALP (U/L) |  | 113 | 183 (33, 1226) | 35 | 203 (51, 453) | 0.996 |
| GGTP (U/L) |  | 102 | 55 (10, 1579) | 35 | 41 (10, 592) | 0.996 |
| Platelet count (x10^3^/μl) |  | 103 | 286 (24, 652) | 35 | 300 (28, 556) | 0.996 |
| APRI |  | 102 | 0.3 (0.1, 37.2) | 34 | 0.3 (0.1, 1.8) | 0.996 |
| FIB-4 |  | 102 | 0.3 (0.1, 16.3) | 33 | 0.3 (0.0, 1.2) | 0.996 |
| Total Immunoglobulin G (IgG) |  | 90 | 1385 (534, 5171) | 30 | 1194 (480, 2064) | 0.134 |
| Positive ANA, ASMA/actin &/or LKM at enrollment |  | 93 | 51 (54.8%) | 31 | 18 (58.1%) | 0.996 |
| Liver Stiffness Median (kPa) |  | 89 | 6.1 (3.0, 63.9) | 26 | 5.7 (3.5, 16.7) | 0.996 |
| Ursodeoxycholic acid |  | 127 | 68 (53.5%) | 37 | 16 (43.2%) | 0.996 |
| Oral Vancomycin |  | 127 | 31 (24.4%) | 37 | 13 (35.1%) | 0.996 |
| Any immunosuppressant |  | 127 | 69 (54.3%) | 37 | 18 (48.6%) | 0.996 |
| Any immunobiological therapy |  | 127 | 46 (36.2%) | 37 | 11 (29.7%) | 0.996 |

* Fisher’s exact or Wilcoxon rank sum test, adjusted for multiple comparisons using the adaptive step-up Bonferroni method.

**SDC Table 7B. Comparison of Autoantibody Positive vs. Negative**

|  | | **No Positive Antibody (N=55)** | | **Positive ANA, ASMA/actin &/or LKM at enrollment (N=69)** | |  |
| --- | --- | --- | --- | --- | --- | --- |
| **Variable** |  | **N** | **n (%) or median (range)** | **N** | **n (%) or median (range)** | **P-value*** |
| Diagnosis | Large Duct PSC | 55 | 42 (76.4%) | 69 | 51 (73.9%) | 0.972 |
|  | Small Duct Only PSC |  | 13 (23.6%) |  | 18 (26.1%) |  |
| AIH at enrollment |  | 44 | 11 (25%) | 55 | 23 (41.8%) | 0.972 |
| IBD at enrollment |  | 55 | 33 (60%) | 69 | 57 (82.6%) | 0.119 |
| Age at consent (years) |  | 55 | 16.1 (3.9, 22.6) | 69 | 16.7 (2.5, 23.7) | 0.972 |
| Sex | Female | 54 | 17 (31.5%) | 69 | 31 (44.9%) | 0.972 |
|  | Male |  | 37 (68.5%) |  | 38 (55.1%) |  |
| Ethnicity | Hispanic | 49 | 2 (4.1%) | 63 | 7 (11.1%) | 0.972 |
|  | Non-Hispanic |  | 47 (95.9%) |  | 56 (88.9%) |  |
| Race | Asian | 50 | 1 (2%) | 63 | 3 (4.8%) | 0.972 |
|  | Black or African American |  | 2 (4%) |  | 4 (6.3%) |  |
|  | Multiracial |  | 2 (4%) |  | 0 (0%) |  |
|  | Native American/Pacific Islander |  | 0 (0%) |  | 0 (0%) |  |
|  | White |  | 45 (90%) |  | 56 (88.9%) |  |
| Length of PSC diagnosis (years) |  | 55 | 1.9 (0.1, 8.3) | 69 | 2.5 (0.1, 10.0) | 0.972 |
| Total bilirubin (mg/dL) |  | 48 | 0.6 (0.2, 2.3) | 63 | 0.6 (0.3, 3.7) | 0.972 |
| AST (U/L) |  | 53 | 35 (14, 727) | 67 | 37 (13, 733) | 0.972 |
| ALT (U/L) |  | 54 | 34 (10, 816) | 67 | 41 (8, 756) | 0.972 |
| ALP (U/L) |  | 52 | 183 (60, 602) | 65 | 187 (51, 1226) | 0.972 |
| GGTP (U/L) |  | 51 | 55 (12, 524) | 58 | 39 (10, 1579) | 0.972 |
| Platelet count (x10^3^/μl) |  | 47 | 301 (28, 537) | 64 | 276 (24, 607) | 0.972 |
| APRI |  | 46 | 0.3 (0.1, 6.0) | 63 | 0.3 (0.1, 37.2) | 0.972 |
| FIB-4 |  | 46 | 0.3 (0.1, 2.2) | 63 | 0.3 (0.0, 16.3) | 0.972 |
| Total Immunoglobulin G (IgG) |  | 48 | 1230 (644, 2560) | 67 | 1430 (480, 5171) | 0.308 |
| Liver Stiffness Median (kPa) |  | 41 | 5.9 (3.0, 63.9) | 54 | 5.8 (3.2, 43.7) | 0.972 |
| Ursodeoxycholic acid |  | 55 | 30 (54.5%) | 69 | 30 (43.5%) | 0.972 |
| Oral Vancomycin |  | 55 | 12 (21.8%) | 69 | 24 (34.8%) | 0.972 |
| Any immunosuppressant |  | 55 | 29 (52.7%) | 69 | 42 (60.9%) | 0.972 |
| Any immunobiological therapy |  | 55 | 16 (29.1%) | 69 | 26 (37.7%) | 0.972 |

* Fisher’s exact or Wilcoxon rank sum test, adjusted for multiple comparisons using the adaptive step-up Bonferroni method.

**SDC Table 7C. Comparison of with/without IBD**

|  | | **No IBD (N=44)** | | **IBD (N=124)** | |  |
| --- | --- | --- | --- | --- | --- | --- |
| **Variable** |  | **N** | **n (%) or median (range)** | **N** | **n (%) or median (range)** | **P-value*** |
| Diagnosis | Large Duct PSC | 44 | 33 (75%) | 124 | 96 (77.4%) | 0.993 |
|  | Small Duct Only PSC |  | 11 (25%) |  | 28 (22.6%) |  |
| AIH at enrollment |  | 37 | 14 (37.8%) | 94 | 31 (33%) | 0.993 |
| Age at consent (years) |  | 44 | 15.8 (4.2, 20.8) | 124 | 16.2 (2.5, 25.6) | 0.993 |
| Sex | Female | 44 | 17 (38.6%) | 122 | 50 (41%) | 0.993 |
|  | Male |  | 27 (61.4%) |  | 72 (59%) |  |
| Ethnicity | Hispanic | 37 | 2 (5.4%) | 115 | 10 (8.7%) | 0.993 |
|  | Non-Hispanic |  | 35 (94.6%) |  | 105 (91.3%) |  |
| Race | Asian | 39 | 1 (2.6%) | 115 | 4 (3.5%) | 0.993 |
|  | Black or African American |  | 5 (12.8%) |  | 5 (4.3%) |  |
|  | Multiracial |  | 1 (2.6%) |  | 1 (0.9%) |  |
|  | Native American/Pacific Islander |  | 0 (0%) |  | 1 (0.9%) |  |
|  | White |  | 32 (82.1%) |  | 104 (90.4%) |  |
| Length of PSC diagnosis (years) |  | 44 | 1.7 (0.1, 8.8) | 123 | 2.6 (0.1, 14.5) | 0.993 |
| Total bilirubin (mg/dL) |  | 38 | 0.6 (0.3, 4.2) | 101 | 0.5 (0.2, 62.0) | 0.993 |
| AST (U/L) |  | 41 | 43 (13, 677) | 111 | 34 (13, 733) | 0.946 |
| ALT (U/L) |  | 40 | 46 (15, 1460) | 112 | 35 (8, 816) | 0.993 |
| ALP (U/L) |  | 40 | 188 (52, 545) | 108 | 189 (33, 1226) | 0.993 |
| GGTP (U/L) |  | 40 | 57 (12, 1193) | 97 | 46 (10, 1579) | 0.993 |
| Platelet count (x10^3^/μl) |  | 38 | 289 (78, 483) | 100 | 292 (24, 652) | 0.993 |
| APRI |  | 38 | 0.4 (0.1, 5.1) | 98 | 0.3 (0.1, 37.2) | 0.770 |
| FIB-4 |  | 37 | 0.3 (0.1, 2.8) | 98 | 0.3 (0.0, 16.3) | 0.993 |
| Total Immunoglobulin G (IgG) |  | 34 | 1304 (534, 5171) | 86 | 1338 (480, 4740) | 0.993 |
| Positive ANA, ASMA/actin &/or LKM at enrollment |  | 34 | 12 (35.3%) | 90 | 57 (63.3%) | 0.127 |
| Liver Stiffness Median (kPa) |  | 31 | 7.0 (4.0, 60.0) | 84 | 5.8 (3.0, 63.9) | 0.351 |
| Ursodeoxycholic acid |  | 43 | 28 (65.1%) | 121 | 56 (46.3%) | 0.801 |
| Oral Vancomycin |  | 43 | 8 (18.6%) | 121 | 36 (29.8%) | 0.993 |
| Any immunosuppressant |  | 43 | 22 (51.2%) | 121 | 65 (53.7%) | 0.993 |
| Any immunobiological therapy |  | 43 | 2 (4.7%) | 121 | 55 (45.5%) | <.001 |

* Fisher’s exact or Wilcoxon rank sum test, adjusted for multiple comparisons using the adaptive step-up Bonferroni method.

**SDC Table 7D. Comparison of with/without IBD and/or AIH**

|  | | **PSC only (N=37)** | | **PSC/IBD (N=93)** | | **PSC/AIH (N=14)** | | **PSC/IBD/AIH (N=31)** | |  |
| --- | --- | --- | --- | --- | --- | --- | --- | --- | --- | --- |
| **Variable** |  | **N** | **n (%) or median (range)** | **N** | **n (%) or median (range)** | **N** | **n (%) or median (range)** | **N** | **n (%) or median (range)** | **P-value*** |
| Diagnosis | Large Duct PSC | 37 | 29 (78.4%) | 93 | 76 (81.7%) | 14 | 11 (78.6%) | 31 | 20 (64.5%) | 0.903 |
|  | Small Duct Only |  | 8 (21.6%) |  | 17 (18.3%) |  | 3 (21.4%) |  | 11 (35.5%) |  |
| Age at consent (years) |  | 37 | 15.7 (4.2, 20.8) | 93 | 16.0 (2.5, 25.6) | 14 | 16.1 (11.7, 20.0) | 31 | 17.7 (7.3, 21.5) | 0.903 |
| Sex | Female | 36 | 13 (36.1%) | 91 | 35 (38.5%) | 14 | 6 (42.9%) | 31 | 15 (48.4%) | 0.903 |
|  | Male |  | 23 (63.9%) |  | 56 (61.5%) |  | 8 (57.1%) |  | 16 (51.6%) |  |
| Ethnicity | Hispanic | 29 | 2 (6.9%) | 86 | 8 (9.3%) | 13 | 0 (0%) | 29 | 2 (6.9%) | 0.903 |
|  | Non-Hispanic |  | 27 (93.1%) |  | 78 (90.7%) |  | 13 (100%) |  | 27 (93.1%) |  |
| Race | Asian | 32 | 2 (6.3%) | 85 | 4 (4.7%) | 13 | 0 (0%) | 30 | 0 (0%) | 0.544 |
|  | Black or African Amer |  | 3 (9.4%) |  | 2 (2.4%) |  | 3 (23.1%) |  | 3 (10%) |  |
|  | Multiracial |  | 1 (3.1%) |  | 0 (0%) |  | 0 (0%) |  | 1 (3.3%) |  |
|  | Native American/ Pacific Isl. |  | 0 (0%) |  | 1 (1.2%) |  | 0 (0%) |  | 0 (0%) |  |
|  | White |  | 26 (81.3%) |  | 78 (91.8%) |  | 10 (76.9%) |  | 26 (86.7%) |  |
| Length of PSC diagnosis (years) |  | 31 | 1.6 (0.1, 8.0) | 92 | 2.2 (0.1, 8.9) | 14 | 1.8 (0.2, 8.8) | 31 | 4.3 (0.1, 14.5) | 0.482 |
| Total bilirubin (mg/dL) |  | 26 | 0.7 (0.3, 4.2) | 76 | 0.5 (0.2, 62.0) | 12 | 0.6 (0.4, 2.3) | 25 | 0.7 (0.2, 2.5) | 0.903 |
| AST (U/L) |  | 28 | 40 (13, 677) | 81 | 35 (13, 733) | 13 | 45 (20, 188) | 30 | 30 (16, 240) | 0.903 |
| ALT (U/L) |  | 28 | 44 (15, 1460) | 81 | 36 (10, 816) | 12 | 57 (15, 253) | 31 | 32 (8, 577) | 0.903 |
| ALP (U/L) |  | 28 | 189 (66, 545) | 79 | 201 (51, 1226) | 12 | 186 (52, 539) | 29 | 144 (33, 578) | 0.903 |
| GGTP (U/L) |  | 27 | 55 (12, 1193) | 71 | 53 (10, 870) | 13 | 59 (15, 327) | 26 | 40 (13, 1579) | 0.903 |
| Platelet count (x10^3^/μl) |  | 25 | 300 (92, 483) | 73 | 301 (24, 652) | 13 | 275 (78, 481) | 27 | 268 (28, 556) | 0.818 |
| APRI |  | 25 | 0.4 (0.1, 4.9) | 72 | 0.3 (0.1, 37.2) | 13 | 0.5 (0.1, 5.1) | 26 | 0.3 (0.1, 5.4) | 0.903 |
| FIB-4 |  | 25 | 0.3 (0.1, 2.8) | 72 | 0.3 (0.0, 16.3) | 12 | 0.4 (0.2, 2.2) | 26 | 0.4 (0.1, 3.8) | 0.903 |
| Total Immunoglobulin G (IgG) |  | 23 | 1269 (534, 5171) | 65 | 1300 (480, 4740) | 11 | 1598 (1050, 3329) | 21 | 1534 (902, 2710) | 0.903 |
| Positive ANA, ASMA/actin, LKM |  | 23 | 7 (30.4%) | 67 | 39 (58.2%) | 11 | 5 (45.5%) | 23 | 18 (78.3%) | 0.067 |
| Liver Stiffness Median (kPa) |  | 23 | 5.8 (4.0, 60.0) | 60 | 5.8 (3.4, 63.9) | 8 | 10.7 (7.0, 25.8) | 24 | 5.8 (3.0, 43.7) | 0.079 |
| Ursodeoxycholic acid |  | 29 | 16 (55.2%) | 90 | 36 (40%) | 14 | 12 (85.7%) | 31 | 20 (64.5%) | 0.021 |
| Oral Vancomycin |  | 29 | 8 (27.6%) | 90 | 24 (26.7%) | 14 | 0 (0%) | 31 | 12 (38.7%) | 0.266 |
| Any immunosuppressant |  | 29 | 10 (34.5%) | 90 | 47 (52.2%) | 14 | 12 (85.7%) | 31 | 18 (58.1%) | 0.099 |
| Any immunobiologic |  | 29 | 1 (3.4%) | 90 | 42 (46.7%) | 14 | 1 (7.1%) | 31 | 13 (41.9%) | <.001 |

* Fisher’s exact or Wilcoxon rank sum test, adjusted for multiple comparisons using the adaptive step-up Bonferroni method.
